# Influenza and respiratory syncytial virus screening for the detection of asymptomatically infected patients in hematology and oncology

**DOI:** 10.3205/dgkh000314

**Published:** 2018-09-24

**Authors:** Claas Baier, Christin Linderkamp, Andreas Beilken, Felicitas Thol, Michael Heuser, Ella Ebadi, Tina Ganzenmueller, Albert Heim, Franz-Christoph Bange

**Affiliations:** 1 Institute for Medical Microbiology and Hospital Epidemiology, Hannover Medical School (MHH), Hannover, Germany; 2 Department of Pediatric Hematology and Oncology, Hannover Medical School (MHH), Hannover, Germany; 3 Department of Hematology, Hemostasis, Oncology and Stem Cell Transplantation, Hannover Medical School (MHH), Hannover, Germany; 4 Institute of Virology, Hannover Medical School (MHH), Hannover, Germany

**Keywords:** PCR, screening, respiratory syncytial virus, influenza, infection control, cross infection, oncology

## Abstract

**Introduction:** Respiratory syncytial virus (RSV) and influenza virus infections are a significant healthcare risk for immunocompromised patients. In addition to community onset, nosocomial acquisition and transmission may also occur. Detection of asymptomatic shedders (e.g., patients in the incubation period or immunosuppressed long term shedders) facilitates control of nosocomial transmission.

**Methods:** To strengthen the existing infection control concept, a PCR-based screening for RSV and influenza virus was implemented for all patients lacking respiratory symptoms (asymptomatic patients) who were hospitalized on an adult and a pediatric hemato-oncological ward. Laboratory results of this screening were analyzed retrospectively.

**Results:** 665 respiratory specimens were obtained for screening from 251 patients (26% were 18 years and younger) from December 2016 to April 2017. In 23 patients without respiratory symptoms, either influenza virus or RSV infection was found, resulting in a detection rate of about 9%. In 6 patients, the infection was presumably detected during the incubation period, because an increase of viral load was observed in subsequent specimens. Positive screening results facilitated timely implementation of adequate infection control precautions. Nosocomial clusters of RSV or influenza were not detected during the screening period on the two wards.

**Conclusion:** The seasonal screening program expanded our existing infection control concept in terms of patients lacking respiratory symptoms who shed influenza virus or RSV. It enabled us to identify 23 RSV or influenza infections in patients lacking respiratory symptoms in a 4-month period and thus to rapidly take isolation precautions.

## Zusammenfassung

**Einleitung:** Infektionen mit dem Respiratory Syncytial Virus (RSV) und dem Influenzavirus stellen für immunsupprimierte Patienten ein relevantes Gesundheitsrisiko dar. Die Infektion mit diesen Viren erfolgt häufig außerhalb des Krankenhauses, jedoch sind auch ein nosokomialer Erwerb und nosokomiale Übertragungen möglich. Die rechtzeitige Detektion von asymptomatischen Virusausscheidern (z.B. Patienten in der Inkubationsphase oder asymptomatische Langzeitausscheider bei Immunsuppression) kann dazu beitragen, die nosokomiale Verbreitung dieser Erreger zu verhindern.

**Methoden:** Es wurde ein PCR-basiertes Screening-Programm für RSV und Influenza für alle Patienten ohne Symptome einer respiratorischen Erkrankung (asymptomatische Patienten) etabliert, die auf einer pädiatrischen hämatologisch-onkologischen und einer hämatologisch-onkologischen Station für Erwachsene behandelt wurden. Die Ergebnisse dieses Screening-Programms wurden retrospektiv analysiert.

**Ergebnisse: **Von Dezember 2016 bis April 2017 wurden insgesamt 665 respiratorische Proben von 251 asymptomatischen Patienten (davon waren 26% pädiatrische Patienten) gewonnen. Bei 23 dieser Patienten konnte entweder eine RSV- oder eine Influenzainfektion nachgewiesen werden, was einer Detektionsrate von etwa 9% entspricht. 6 der Patienten, die eine ansteigende Viruslast im Verlauf aufwiesen, befanden sich beim Screening mutmaßlich in der Inkubationsphase. Positive Screening-Ergebnisse führten zur Implementierung zusätzlicher Hygienemaßnahmen (z.B. Isolation, Barrierepflege). Influenza und/oder RSV Häufungen wurden auf den beiden Stationen im Screening-Zeitraum nicht detektiert.

**Schlussfolgerung: **Das saisonale Screeningprogramm hat unser bestehendes Hygienekonzept in der Hämatologie und Onkologie in Hinblick auf asymptomatische Virusausscheider erweitert. Es ermöglichte in einer etwa viermonatigen Periode insgesamt 23 asymptomatisch infizierte Patienten zu detektieren und für diese adäquate Hygienemaßnahmen zu ergreifen.

## Introduction

Influenza virus (family *Orthomyxoviridae*) and respiratory syncytial virus (RSV; family *Pneumoviridae*) are both single-strand RNA viruses and cause upper and lower respiratory tract infections in children and adults [1], [2], [3], [4]. Influenza virus and RSV are spread by droplet and contact transmission. Therefore, nosocomial acquisition may occur due to exposure to another infected patient, visitor or healthcare workers (HCWs), as well as by contact with contaminated, inanimate surfaces. Immunocompromised patients with underlying hemato-oncological disorders or cancer are at risk for increased mortality, especially in the case of lower respiratory tract infection due to these viruses [5], [6], [7]. Both community and nosocomial acquisition of influenza and RSV are possible in this patient group. Nosocomial outbreaks in oncologic care facilities have been described [8], [9] in spite of multiple infection control measures to prevent nosocomial transmission. These measures include stringent hand hygiene, patient isolation, and use of barrier precautions such as surgical masks or vaccination (influenza) [5], [10], [11], [12]. 

During a localized nosocomial cluster with RSV at our clinic in the winter season 2015/2016, we introduced a RT-PCR-based screening of asymptomatic patients for RSV and influenza virus which contributed to the successful control of the RSV cluster [13]. To strengthen our existing infection control measures in hematology and oncology, we subsequently implemented a systematic influenza and RSV screening of patients lacking respiratory symptoms on a pediatric and an adult hemato-oncological ward as a prophylactic infection control measure in the following winter (2016/2017). This screening intended to identify patients shedding RSV or influenza virus without signs of disease and – in case of a positive screening test – to establish infection control measures similar to those used for typical symptomatic (e.g. cough, sneezing, fever, respiratory distress) patients with RSV or influenza. 

In this paper, we describe the laboratory results of this prophylactic screening program for asymptomatic patients (season 2016/2017) and discuss its value for infection control in hematology and oncology. 

## Materials and methods

### Setting

The screening program for RSV and influenza was implemented on two wards located in the Department of Pediatric Hematology and Oncology and in the Department of Hematology, Hemostasis, Oncology and Stem Cell Transplantation at Hannover Medical School. These departments are tertiary referral centers for children and adults, respectively, with hematologic and solid neoplasia. The pediatric ward contains 5 single (one-bed) and 5 two-bed rooms. The adult ward has 4 single (one-bed) rooms, 6 two-bed rooms, and 3 four-bed rooms. All single rooms on the pediatric ward and 3 single rooms on the adult ward have an anteroom and high-efficiency particulate air filtration with the air flow directed to the hallway. Single rooms are usually reserved for patients with severe immunosuppression or palliative settings. The wards are serviced by permanent healthcare workers (HCWs) and housekeeping staff. Parents are allowed to stay overnight with their children on the pediatric ward. During their stay on the ward, parents are permitted to wear street clothes. However, a regular change of their clothing is recommended. 

### Screening and diagnostic tests

The PCR-based seasonal screening for RSV and influenza started in the second half of December 2016 and ended at the beginning of April 2017. The start and finish of the screening period were set jointly by the infection control staff, virologists and the clinicians involved based on available epidemiological data. On the adult ward, the screening was performed upon admission (admission screening) and once weekly (prevalence screening). On the pediatric ward, the PCR-based screening was performed once weekly. Respiratory material was obtained from all patients lacking respiratory symptoms (asymptomatic patients). Prophylactic prevalence screening was continued as long as patients stayed on the wards. Patients with any symptoms of respiratory disease, e.g., cough, sneeze, fever or oxygen need (symptomatic patients), received a full diagnostic virology panel including influenza virus and RSV, and other respiratory viruses such as human metapneumovirus, adenovirus, parainfluenza virus, coronavirus and rhino-/enterovirus. 

In order to guarantee high adherence to the screening program, repeated audits and feedback talks took place. 

### Processing of screening specimens and retrospective data analysis

Specimens were collected as a combined nasopharyngeal swab or pharyngeal lavage (oral wash) with sterile saline and were processed at the Institute of Virology. For real-time RT-PCR, RNA was extracted using a QiaAmp Viral RNA Mini Kit in a QIAcube according to the manufacturer’s instruction (Qiagen, Hilden, Germany). cDNA synthesis, amplification and detection of nucleic acid were performed in an Applied Biosystems^®^ 7500 Real-Time PCR System (Life Technologies, Carlsbad, California, USA) using a commercially available one-step real-time RT-PCR kit (RSV/hMPV r-gene^®^ PCR Kit, bioMérieux, Nürtingen, Germany) according to the manufacturer’s instructions, and an influenza A/B multiplex real time RT-PCR [14]. The influenza RT-PCR differentiates between types A and B. For semi-quantitative comparison of viral loads with follow-up specimens, the cycle threshold (ct) value of the real time PCR was used. A decrease of the ct by ≥4 values (usually equivalent to a more than 10-fold increase of viral load) was considered as a significant increase of viral load. 

### Infection control interventions for asymptomatic patients with RSV or influenza virus, detected by the screening 

Infection control recommendations for asymptomatically infected patients with RSV and influenza virus were the same as for symptomatic patients. They were labeled in an electronic alert system, and it was recommended that the patients each be placed in a single (one-bed) room. Each positive PCR test was electronically communicated to the infection control staff. Contact and droplet precautions (surgical mask, gown, gloves) were mandatory for HCWs and all visitors entering the room of affected patients. The patients were restricted to their single (one-bed) room and were trained in hand hygiene. If they had to leave the room, they wore a surgical mask. Two negative RT-PCR results at a minimum 2-day interval were required to stop all isolation precautions.

HCWs were strongly encouraged to let themselves be vaccinated against influenza before and during the winter season 2016/17. The vaccination rate among HCWs for influenza was about 60% on the pediatric ward and about 30% percent on the adult ward.

## Results

From December 2016 to April 2017, 251 asymptomatic patients were screened. 66 (26%) of these patients were aged 18 or younger and were screened on the pediatric ward. Of the remaining 185 adult patients, 11 (5.9%) received swabs on the emergency ward, whereas 174 patients were screened exclusively on the adult hemato-oncological ward. Overall, 665 screening samples were obtained, 23% of which (n=152) were from pediatric patients and 77% from adults (n=513). The mean screening sample number per patient was 2.3 for pediatric patients and 2.8 for adults. 

The majority (98%) of the screening samples were nasopharyngeal swabs (n=654). 11 samples were pharyngeal washes and were exclusively obtained from adult patients. 

Altogether, 23 asymptomatic patients tested positive either for influenza or RSV in a screening sample, i.e., a combined detection rate of 9%. 11 patients (10 adults and one child) tested positive for influenza type A. 12 patients (5 adults and 7 children) were tested positive for RSV. Co-infections with influenza virus and RSV were not detected. An overview is shown in Table 1 (Tab. 1). Interestingly, in 6 patients (5 influenza virus patients and 1 RSV patient), a significant increase of the viral load in samples was observed following the positive screening specimen (Table 2 (Tab. 2)). 

13 of the 23 (57%) asymptomatic patients were in two- and four-bed rooms at the time the screening test was reported positive (4 positive for RSV, 9 positive for influenza virus). 6 of these patients in shared rooms were immediately moved to separate single rooms, following the infection control recommendations. 2 patients with influenza were kept together, as they occupied the same 2-bed room when they simultaneously tested positive. 2 patients with RSV and 1 patient with influenza stayed in the shared rooms, and 2 patients had already been discharged when the test result became available. 10 of the 23 asymptomatic patients were already located in single (one-bed) rooms (for reasons other than infection control) and remained in this room after virus detection.

For all patients identified (whether located in private or shared rooms after positive test results were available), barrier precautions (droplet and contact) were implemented as described above (see “Materials and methods” section). 

On both wards, all patients presenting with symptoms of acute respiratory infections were routinely tested with a broad panel for respiratory viruses (including RSV and influenza). In these patients with symptoms of acute respiratory infection, we found 1 influenza case and 1 RSV case on the pediatric ward and 1 influenza case and 3 RSV cases on the adult ward from December 2016 to April 2017. Prevalence of other respiratory viruses was low in this season on these two wards. 1 parainfluenza case, 6 human metapneumovirus cases and 4 rhinovirus cases were found on the pediatric ward. On the adult ward 1 parainfluenza case was detected. Clusters of nosocomial transmission of RSV or influenza were not observed during the screening program period.

## Discussion

Respiratory tract infections caused by RSV and influenza virus are a significant healthcare risk for hospitalized and non-hospitalized patients with underlying hemato-oncological disease. During the winter season, acquisition of these viruses typically occurs in the community, and patients may be admitted with symptoms of infection or infection to a hospital. Nosocomial acquisition due to transmission occurs as well [15], [16], [17], [18]. Recommendations and suggestions for controlling the spread of influenza and RSV in healthcare settings in general and especially in hemato-oncological patients exist (e.g. [5], [10]), but should be adapted to each hospital’s specific needs. To extend our existing standard infection control measures, we introduced a prophylactic screening program for asymptomatic patients targeting RSV and influenza on an adult and a pediatric hemato-oncological ward. The screening was intended to detect patients that shed the virus without having signs or symptoms of disease. Shedding my occur before the onset of disease (incubation period), during an asymptomatic clinical course, or following disease as a result of prolonged viral shedding, particularly in the immunocompromised host [19]. These groups of asymptomatic patients, who often remain unnoticed, can contribute to nosocomial spread. Of the 23 asymptomatic shedders, we detected an increased viral load in 6 patients in follow-up specimens (see Table 2 (Tab. 2)). It is likely that these patients were in the incubation period. This group of patients is of particular interest for infection control strategies, since with increasing viral load, the risk of transmission also rises. 

We identified 23 of a total of 251 asymptomatic patients with an RSV or influenza virus infection (detection rate of about 9%).This means that about 11 asymptomatic patients needed to be screened to detect one shedder of either RSV or influenza virus. Other studies found a similar prevalence in asymptomatic individuals for influenza (8.3%) and RSV (4.4%) using RT-PCR [20], [21]. Campbell et al. examined samples from asymptomatic and symptomatic patients prior to hematopoietic stem-cell transplantation (HSCT) in a study to evaluate the clinical outcomes associated with respiratory virus detection before HSCT [22]. During a 5-year period, 308 asymptomatic adult and pediatric patients were screened, but only one patient with RSV and none with influenza virus were identified. Interestingly, this study found other viruses (e.g., human rhinovirus or coronavirus) in 23 patients. These respiratory viruses were not included in our screening program, as they rarely cause severe lower respiratory tract disease. 

13 out of 23 asymptomatic patients who tested positive for RSV or influenza virus in our study occupied shared patient rooms at the time of virus detection. The majority were moved to single rooms (one-bed rooms) or cohorting was used. Droplet and contact precautions were used for all affected patients. Isolation and barrier precautions regarding virus shedders is important, as transmission to roommates has been described [16]. The transfer to single (one-bed) rooms was challenging in some cases, due to the lack of single-room availability. Nonetheless, because of the high mortality rate of RSV and influenza infection in hemato-oncological patients, isolation and barrier precautions were enforced. With these measures, we aimed to lower the risk of patient-to-patient transmission by reducing the exposure of contact patients. The usage of droplet and contact precautions (such as surgical mask and gown) was also intended to reduce exposure of HCWs. All HCWs, whether vaccinated against influenza or not, wore masks when they performed care in patients with RSV or influenza virus. A further benefit of early detection of influenza virus is the possibility of early antiviral therapy for infected patients and prophylaxis with antiviral agents for contact patients [5]. An additional effect of the screening program and the associated audits and feedback talks was the heightened awareness among HCWs and visitors for respiratory viral infections in this highly susceptible, immunocompromised patient population. During the screening period, no nosocomial cluster of influenza virus or RSV was observed on the two wards. 

Our investigation has limitations. First, it relied on virological laboratory data analyzed retrospectively. Further information, such as the clinical course and outcome of the patients identified by screening, was not accessible for this study. We therefore could not correlate the laboratory results with the clinical course of the patients. Viral loads were estimated semi-quantitatively by comparing ct values of real-time PCR between positive screening and follow-up specimens. However, this approach has been established for respiratory specimens by previous studies on influenza and various other pathogens [23], [24], [25], [26].

## Conclusions

Multifaceted approaches are necessary to help to control the nosocomial spread of RSV and influenza in healthcare settings. Infection control is based on standard precautions (e.g., compliance with hand hygiene, cough etiquette, etc.) together with RSV and influenza pathogen-specific precautionary measures (e.g. droplet precautions, isolation). In special high-risk settings such as hematology and oncology units, additional measures can be useful to lower the risk of hospital acquisition of RSV and influenza. Therefore, we implemented a prophylactic RT-PCR-based RSV and influenza screening program targeting asymptomatic patients. This seasonal screening program enabled us to identify 23 patients who lacked symptoms for respiratory disease, but were infected with either RSV or influenza during a period of about 4 months. For these patients the same infection control practices were implemented as for symptomatic patients with RSV and influenza infection. In our view, this screening program proved useful for identifying asymptomatically infected patients with viral shedding, thus reducing the risk of transmission and potential nosocomial clusters of RSV and influenza virus on hemato-oncological wards. Nevertheless, future studies on larger cohorts, including the analyses of clinical data, are necessary to further validate the use of a seasonal screening program as suggested here.

## Notes

### Competing interests

The authors declare that they have no competing interests.

### Ethical approval

We obtained ethical approval for this study from the ethics committee of the Hannover Medical School (Number 3672-2017). 

### Funding

This research did not receive any specific grant from funding agencies in the public, commercial, or not-for-profit sectors.

### Authors’ contributions

All authors contributed to the manuscript according to the ICMJE (International Committee of Medical Journal Editors) recommendations:

AH and CB were responsible for data acquisition. All authors were involved in analysis and interpretation of the data. TG and AH supervised laboratory diagnostics. CB and FCB prepared the manuscript. CB organized the drafting process. CL, AB, MH and FT implemented and supervised the screening program on the wards. All authors critically revised the manuscript and are accountable for accuracy and correctness. 

All authors have read and agreed to the final draft before submission.

### Acknowledgements

We acknowledge the technical laboratory staff for the processing of screening specimens. 

## Figures and Tables

**Table 1:**
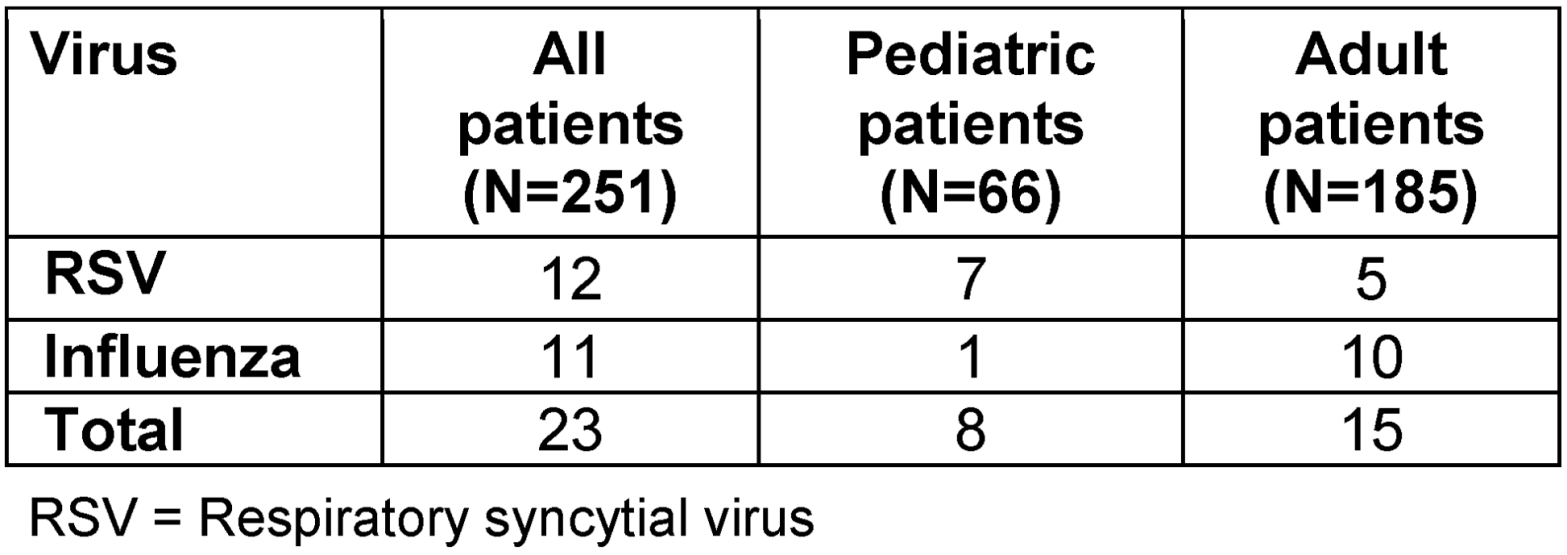
Overview of RT-PCR screening results of patients lacking respiratory symptoms. From December 2016 to April 2017, altogether 251 asymptomatic patients were screened. In 23 patients either influenza or RSV was detected.

**Table 2:**
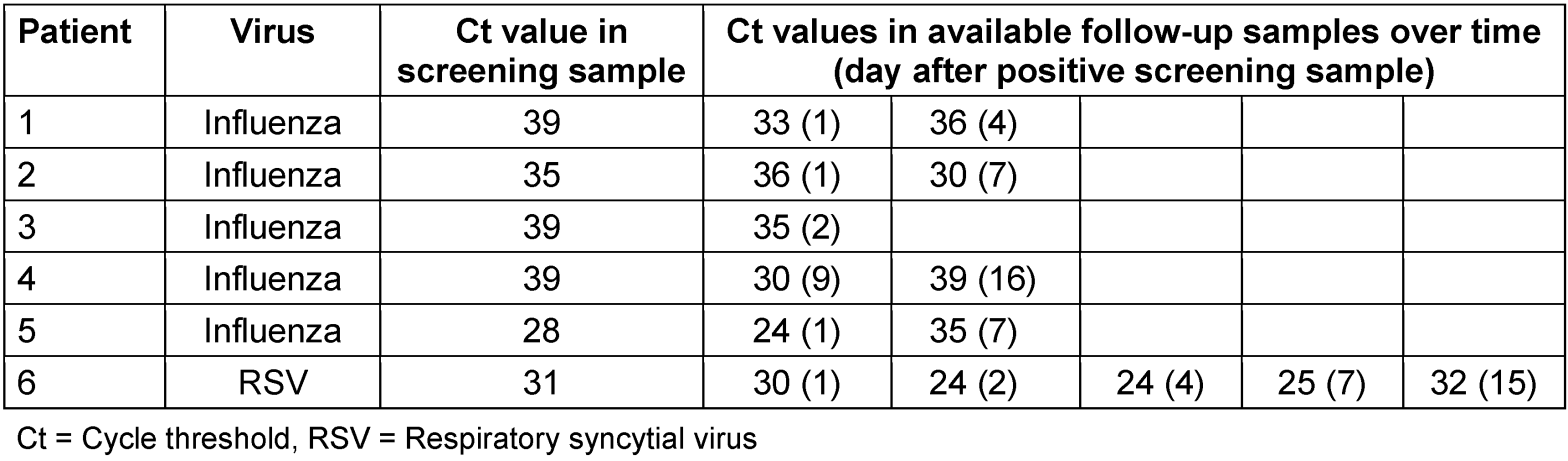
Course of viral load in 6 patients who showed an increase in viral load (presumed detection during the incubation period). The 6 patients showed an increase in viral load during their clinical course and were therefore presumably detected during the incubation period.
